# Patient‐Centred Quality of Nursing Care: A Cross‐Sectional Study of the Level and Associated Factors as Perceived by Patients

**DOI:** 10.1002/nop2.70995

**Published:** 2026-11-01

**Authors:** Helena Leino‐Kilpi, Sanna Mäkinen, Saija Inkeroinen, Miko Pasanen, Jenna Poraharju, Heli Virtanen, Minna Stolt

**Affiliations:** ^1^ Department of Nursing Science University of Turku Turku Finland; ^2^ Wellbeing Services County of Southwest Finland/Turku University Hospital Turku Finland; ^3^ Wellbeing Services County of Satakunta/Central Hospital Pori Finland

**Keywords:** Good Nursing Care Scale, patient‐centred quality of nursing care, quality of care

## Abstract

**Aim:**

To produce evaluative knowledge on the patient‐centred quality of nursing care and factors related to it from the perspective of hospitalized patients in Finland.

**Design:**

A cross‐sectional explorative study.

**Review Methods:**

Survey instrument included the structured, internationally validated Good Nursing Care Scale (40 items) (Leino‐Kilpi 2025), General Evaluation of the Quality of Nursing Care (5 items) and background factors (15 items). The data were statistically analysed.

**Data Sources:**

Data were collected between May and July 2025 from hospitalized adult patients (*N* = 861), from two wellbeing services counties in Finland.

**Results:**

Patients evaluated the quality of nursing care high: subcategories of Good Nursing Care Scale (Median 3.80–3.00, Scale 1–4), The General Evaluation of the Quality of Nursing (Median 9, Scale 4–10) and Patient educational support (Median 3.46, Scale 1–4). Lowest ratings were in the collaboration with the family/significant others. Identified statistically associated factors were patients´ work status and health status on the day of discharge, and sufficient information prior hospitalization.

**Conclusion:**

The results both confirm existing knowledge and identify development needs in conceptual, methodological and clinical fields.

**Implications for the Profession and Patient Care:**

The research has implications both on the patient and organizational levels, including role of the patients and leadership of nursing quality.

**Impact:**

The public social and healthcare reform underscores the need for quality evaluation in Finland. The research showed the patient‐centred quality of nursing care in hospital being high, but identified areas for development. The findings may impact on nursing practice, nursing education and healthcare organizations.

**Reporting Method:**

Reported in accordance with the STROBE guidelines.

**Patient or Public Contribution:**

Patients were voluntary respondents in the study.

## 1 Introduction

Patient‐centred nursing care is globally recognized as an effective method for developing nursing quality by respecting the values, needs and preferences of patients (Havana et al. 2023). Evaluating the quality of patient‐centred nursing care is part of health care quality assurance and should be continually evaluated to monitor progress as well as areas of development. This requires systematic use of valid instruments and timely data.

In recent years, patient experiences—along with evidence‐based care, patient engagement and sustainable development goals—have become central to health care evaluations and quality development (Kendir et al. 2022; OECD 2025a). Most existing research (Attar and Shukla 2025; Jamieson Gilmore et al. 2023) in the field of patient‐reported experience measures (PREMS) has focused on the micro level of care (patient‐level), developing quality of front‐line patient care. On the meso‐level (organizational), these measures have been used in quality‐based financing or in performance development. Few macro‐level (health system‐level) uses are reported.

Currently, national and international reforms of health care organizations, changes in the role of patients and challenges related to nurses' workload and shortages require continuous data related to quality of patient‐centred nursing care (Havana et al. 2023; Navarro et al. 2021; Volakakis et al. 2024; WHO [World Health Organization] 2026). In this study, we produce multidimensional patient‐reported experiential knowledge about the quality during the nursing process (PREMS) and analyse the potential association of the quality with individual sociodemographic factors and factors related to care and health of the patients. As such, the study responds to the current international research need in the field.

## 2 Background

Patient‐centred quality of nursing care has been defined in various ways. In this study, we adopt an action‐based definition of nursing, conceptualizing nursing as an intentional human action (Mattila et al. 2025; Eyster et al. 2022). This definition emphases the importance of the actor (nurse), activities, preconditions, proceeding of the nursing process, environment, patient empowerment and collaboration with significant others. Based on this definition of nursing, Good Nursing Care Scale was used and it has demonstrated applicability across different countries over an extended period (Mattila et al. 2025), and can be combined with other instruments (Correia et al. 2025; Guzmán‐Leguel and Rodríguez‐Lara 2025). Nursing is a key component of health care quality and in Finland, the number of practising nurses per population is among the highest in Organization for Economic Co‐operation and Development (OECD) countries, as is the nurse‐to‐doctor ratio (EU Healthcare 2026; OECD 2025b).

Although variation can be found across countries and clinical contexts, earlier studies generally show that patients' evaluations of the quality of nursing care are mainly positive (Havana et al. 2023). Even so, some challenges have been identified. Care quality has been reported to be lower in situations where each nurse is responsible for a high number of patients (high patient‐to‐nurse ratio), poor work environments for nurses, insufficient physical care, patient education or preparation for homecare and limited collaboration with significant others (Guzmán‐Leguel and Rodríguez‐Lara 2025; Mattila et al. 2025; Navarro et al. 2021). Background factors are individual factors related to the sociodemographics of patients, and factors related to nursing care and health of the patient. These factors have been identified as important for patient‐centred quality; however, findings across various contexts are inconsistent. The significance of age, educational background, living arrangements, work status, hospital admission and length of hospital stay can vary between studies (Guan et al. 2024; Mattila et al. 2025; Volakakis et al. 2024). In contrast, good health status on the day of discharge and knowledge about one's care have been more reliably identified as elements developing quality of care (Mattila et al. 2025). Experienced quality also seems to be associated with national health expenditure per capita as well as other factors beyond the health system (OECD 2025a). Although some research has also found that knowledge and education among patients are associated with how patients experience care quality (Inkeroinen et al. 2025), other results contradict this association (Lyu et al. 2024). Despite these inconsistencies, however, there seems to be a need to develop patient education, particularly in psychosocial, ethical and financial aspects of care (Arffman et al. 2025).

This study focused on generating knowledge about the level of nursing care quality and its associated factors, based on hospitalized patients' evaluations in Finland. Patients in this context have the legal right to good health care, and their perspectives on their care are emphasized (Ministry of Social Affairs and Health 2024; OECD and European Observatory on Health Systems and Policies 2025). Further, a large reform integrating social and health care services was implemented in 2023, after the COVID‐19 pandemic (2020–2023).

## 3 The Study

### 3.1 Aim and Objective

This cross‐sectional explorative study aims to generate new evaluative experiential knowledge of the quality of hospital nursing care, from the perspective of hospitalized patients in Finland. Specifically, our objective was to identify both the level of quality of nursing care and its associated factors (sociodemographics and those related to care) in order to identify potential quality gaps and future development needs. The knowledge produced may inform patients, nursing practitioners, leaders, teachers and researchers.

### 3.2 Research Questions

What is the level of the patient‐centred quality of nursing care as perceived by hospitalized patients?

What factors, if any, are associated with the level of the patient‐centred quality of nursing care?

## 4 Methods

### 4.1 Design

This is an explorative cross‐sectional study based on data produced through written self‐evaluations of patients in hospital care.

### 4.2 Instrument

Data were collected using the current version of structured Good Nursing Care Scale (GNCS, Leino‐Kilpi 2025), a 40‐item instrument used to evaluate the quality of nursing care from the patient's perspective. Not any changes were made in the instrument. This scale, originally developed in the 1990s, has been updated several times and has been translated into 10 languages. Its validity and reliability have been established in both national and international contexts (Mattila et al. 2025), including through a Rasch analysis (Stolt et al. 2019). The scale is based on the action‐theory approach to nursing, and it includes the following subcategories of the quality of nursing: nurse characteristics (actor), nursing care activities, preconditions for care, nursing environment, proceeding of the nursing process, patient empowerment and collaboration with family members/significant others. Each subcategory includes 5–7 items and is scored on a 4‐point scale: 4 = completely agree, 3 = almost agree, 2 = almost disagree, 1 = completely disagree, 0 = I cannot evaluate this item. The level of nursing care quality can be interpreted as follows: mean 1–1.5 = very low quality, 1.6–2.0 = low, 2.1–2.5 = fairly low quality, 2.6–3.0 = fairly high quality, 3.1–3.5 = high and 3.6–4.0 = very high quality (Mattila et al. 2025). The primary outcome of the study is the quality of nursing care, expressed by the Summative GNCS score and subcategories of the GNCS are detailed parts of the primary outcome. Added to the subcategories, a new EDUSupport variable was constructed, including the items in subcategories related to educational support of the patient. This new variable has the same purpose as the subcategories: it is detailed part of the primary outcome.

In addition to the GNCS, data were collected using the General Evaluation of the Quality of Nursing Care (GEQN), with five items: quality of nursing care as a whole, patient‐centredness, fluency, effectiveness and competence of nurses. These items were evaluated on a scale of 4–10 (lower value indicating lower level of quality of nursing care). GEON was used as an explorative variable for analysing the summative level of the quality of nursing care. Furthermore, 15 items relating to sociodemographics and to nursing care and health of the patients.

### 4.3 Population, Sample and Data Collection

The data were collected among patients in hospital care (*N* = 861) in Finland in May–July 2025. In the reform of the public health care and social welfare sector in 2023, 21 wellbeing services counties were established to function alongside the independently acting City of Helsinki. All these counties were given responsibility for the public health and social care of their residents as well as the organization of fire and rescue services, under the Ministry of Health and Social Affairs. In the counties, health centres have the main responsibility for primary care and hospitals for specialized care, which is organized into five university‐affiliated collaborative areas for healthcare and social welfare (EU Healthcare 2026).

In this study, a patient sample, drawn from two wellbeing services counties (County 1 and County 2) and the main hospital in both of these counties was used. Both of the counties belong to the same university‐affiliated collaborative area. The counties were selected purposefully, including main clinical fields and—in terms of quality of care and treatment—both counties are required to carry out self‐monitoring and have established systems for patient feedback. Thus, they represent a standard wellbeing county. Together, these counties cover more than 700,000 inhabitants and provided a range of social and health services, hospital services, services for the elderly and rescue and emergency services (Statistics Finland 2026). There are about 35,000 employees working in these counties, nurses being the largest group (Finnish Institute for Health and Welfare 2026).

The data were collected from patients on the day of hospital discharge (either to home or to another institution). To be included, patients had to be at least 16 years old, have had a hospital stay of at least 2 days, be able to self‐administer the instrument in Finnish and voluntary participation based on informed consent. In the hospitals, patients from two main specialities were included: operative (abdominal, cancer, gynaecological, heart, neurology, orthopaedics and traumatology) and medical units (acute, internal, haematology, lung and skin diseases). The sample size was based on a power analysis of the GNCS (Mattila et al. 2025), which indicated a minimum of 300 patients in both counties. In the planning meetings with nurses, however, uncertainties related to organizational changes were identified, and data collection was limited to a fixed time and based on the statistics of patient discharges. Consequently, 40–80 patients per unit were invited, which meant a total of 1300 invited patients. Out of them, 861 (= *N*) responded (66%).

An information session was arranged in each unit informing the contact persons/nurse managers about the purpose of the study, the procedure of data collection and informed consent. The contact persons (primarily ward managers) maintained communication with the researchers throughout the data collection period. Patients responded to the instrument either online or using a paper‐based format. Paper responses were returned in a closed envelope in a box on the ward, and online responses were collected with REDCap (Research Electronic Data Capture) hosted by University of Turku. Patients were advised to respond independently, without discussion with other patients in the room or significant others. If they experienced any physical problems in writing (e.g., due to an orthopaedic operation) or in eyesight, they could receive practical help from a significant other.

### 4.4 Data Analysis

Data were analysed statistically using R version 4.3.0. Descriptive statistics were calculated to characterize the sample. Several background variables were recoded prior to analysis. Work status was collapsed into two categories: working (employed) and not working (retired, unemployed, student or stay‐at‐home parent). Education level was reduced to comprehensive/secondary versus university/polytechnic/doctoral degrees, and current health status was grouped as excellent/good versus fairly good/poor. Time of hospital admission information, originally collected as an open‐ended response, was manually categorized as emergency, 1–3 days ago, 4–10 days ago, 11–30 days ago, or over a month ago, while the open‐ended response on length of hospitalization was categorized as ≤ 2, 3–5, 6–10, and ≥ 11 days. Units were collapsed into medical and operational based on their function and organizational structure.

Seven sum variables were formed based on the structure of the GNCS instrument. The sum variable Collaboration with family members/significant others was found to be distinct from the other subcategories in a Spearman correlation analysis, so it was left as its own variable and Summative level of GNCS. Responses of ‘I cannot evaluate’ on GNCS items were treated as missing data. To allow for incomplete responses, a maximum of one missing item per sum variable was permitted, and for the Summative level of GNCS score, a maximum of two sum variables with one missing item each was allowed. A new sum variable, Educational Support (EDUsupport), was created by summing 13 GNCS items including educational content in all other subcategories of GNCS except Nursing environment (see Table 4). This EDUsupport‐variable was created for producing deeper understanding of the quality of the category of nursing activities, more exactly patient education, a central element in the patient‐centred quality of nursing care (e.g., Arffman et al. 2025; Lyu et al. 2024). Thus, the new EDUsupport is an analytical adding to the evaluation of the quality of nursing activities and would add the relevance of the use of the GNCS in the evaluation of nursing care.

Furthermore, an Alternative summative GNCS score, excluding patient empowerment, was calculated to examine the relationship between patient empowerment, the remaining subcategories and the Alternative summative GNCS score. This was also created for producing a deeper understanding of the empowerment of patients and support of it, as a quality category. Empowerment is a multidimensional concept with many different definitions and expresses both the process and outcome of the quality of nursing care from the perspective of patients' own power, capacity, decision making and management of their own health (Pekonen et al. 2020). Professionals can support the empowerment of patients (Leino‐Kilpi et al. 1999) and in this study, the subcategory of the quality is mainly related to the support of empowerment as experienced by patients. The potential association with the Alternative summative GNCS score could inform about the importance of patient‐related empowerment in the development of the quality of nursing care.

Statistical testing between selected background variables and the Summative level of GNCS, subcategories, the General Evaluation of Quality of Nursing care (GEQN) and EDUsupport were done using the Mann–Whitney *U* test, the Kruskal–Wallis test with Dunn's test for pairwise testing using Bonferroni correction, and Spearman correlations. Visual inspection of the data indicated non‐normal distributions; therefore, non‐parametric tests were used. To limit the risk of inflated Type I error from multiple testing, the subcategories of GNCS were analysed only for background variables with *p* < 0.1 in the Summative level GNCS test. The threshold was slightly relaxed from *p* < 0.05 to avoid missing potentially relevant subcategory effects. To account for multiple testing, the Holm–Bonferroni correction was used to control family‐wise error rate. The Holm–Bonferroni adjustment was applied to the Summative level of GNCS as one family, and as well separately to each set of subcategory analyses. Effect sizes were calculated to assess the influence of the background variables on the GNCS and its subcategories, using *r* for the Mann–Whitney *U* tests and *η* ^2^ for the Kruskal–Wallis tests; 95% confidence intervals were estimated using bootstrapping. *p*‐values < 0.05 were considered statistically significant. Internal consistency of the instrument was assessed using omega coefficients.

For multivariate analysis, linear regression was used the Summative level of GNCS as output variable and factors related to the process of care and health of the patient as inputs. In the process of care, from the beginning to the end, following variables were included: received sufficient information prior to hospitalization, type of admission into hospital, first time at hospital generally, knowledge about proceeding of care and treatment and days spent in this hospital/unit. In the health of the patient, two variables were used: any long‐term disease, current health status. Regression diagnostics indicated some potentially influential observations but sensitivity analysis showed no substantial changes in estimates. Residual normality and homoscedasticity, as well as VIF, were also assessed and deemed acceptable.

### 4.5 Ethical Considerations

Ethical guidelines of research were followed throughout the entire research process (ALLEA 2023). Ethical approval was granted by the Ethical Committee of the University Turku (18th of March 2025, TY/253/2025) and permissions were obtained from both wellbeing services counties (County 1: 27th of March 2025 and County 2: 24th of April 2025). Patients participated voluntarily and provided written informed consent at the beginning of the instrument.

Permission to use the GNCS (Leino‐Kilpi, 2025) was obtained from the copyright holder.

## 5 Results

### 5.1 Subjects

The sample consisted of 861 patients (*N* = 861, Table 1) with an average age of 63 years (range 16–94 years). Most participants had completed secondary school as their highest level of education. The majority (62%) were retired and living with a family member or significant other.

**TABLE 1:** Description of the subjects (*N* = 861).

Variable	*n*	%	Mean (SD)	Median (IQR)	Min–Max
*Sociodemographics*					
Age (years)	847		63 (16)	67 (20)	16–94
Gender	853				
Male	435	51			
Female	416	49			
Does not want to disclose	2				
Education	837				
Comprehensive school	223	27			
Secondary education	346	41			
Bachelor level university/polytechnic degree	154	18			
Master level university/polytechnic degree	110	13			
Doctoral degree	4				
Work status^b^					
Retired	533	62			
Employed	265	31			
Unemployed	56	7			
Student	22	3			
Stay‐at‐home parent	4	1			
Living arrangement	853				
Live alone	283	33			
Live with family members/significant others	570	67			
*Current hospitalization*					
Wellbeing services county	852				
1	325	38			
2	527	62			
Type of admission into hospital	849				
Emergency patient	504	59			
Elective patient	345	41			
Information of admission into hospital (days)	318				
1–3	30	4			
4–10	46	6			
11–30	82	10			
Over a month	160	19			
Received sufficient information prior to hospitalization	830				
Yes	606	73			
No	224	27			
Prior information from^b^					
Health centre nurse/physician	265	31			
Private nurse/physician	166	19			
Elsewhere	140	16			
Sought themselves	94	11			
Relatives/friends	42	5			
Occupational health care	36	4			
First time at hospital generally	851				
Yes	100	12			
No	751	88			
Number of earlier hospitalizations in this hospital	637				
1	2				
2–5	390	61			
6–9	116	18			
> 10	129	20			
Reason for current hospitalization^b^					
An examination	423	49			
Surgical treatment/procedure	433	50			
Medication and/or infusion therapy	370	43			
Guidance/counselling visit due to the illness	98	11			
Other	46	5			
Any long‐term disease	854				
Yes	606	71			
No	248	29			
Type of the long‐term disease^a^					
Heart and vascular diseases	420				
Diabetes	146				
Asthma and allergies	118				
Digestive and endocrine diseases	106				
Musculoskeletal diseases	59				
Cancer	51				
Neurological diseases	29				
Current health status in comparison with normal state of health	847				
Excellent	32	4			
Good	286	34			
Fair	458	54			
Poor	71	8			
Days spent in this hospital/unit	820				
2	248	30			
3–5	359	44			
6–10	149	18			
11 or more	64	8			
Knowledge about proceeding of care and treatment	852				
Yes	736	86			
No	116	14			

*Note:* Percentages were rounded to the nearest whole number, values less than 1% were omitted.

^a^ Patients could report multiple diseases.

^b^ Multichoice.

More than half of the patients were admitted in an emergency situation (59%), while 41% were elective patients, with reasons for hospitalization varying. Adult patients were recruited from somatic units, divided in surgical and medical ones, according to the organizational structures. In operative units, the most common reason for admission was surgical operation (50%), whereas in the medical units it was for an examination (49%) or medication/infusion therapy (43%). The time from when patients had been notified of hospital admission ranged between the same day and over 1 month prior. The majority (73%) of the patients evaluated the information received prior to hospitalization as appropriate; and the information had been received either from a physician or a nurse at the health centre. The patients' length of stay in hospital was mostly 3–5 days (range 2–100 days), and for 12%, this was their first hospitalization.

Regarding health status, 71% of patients had a long‐term disease, most commonly heart and vascular disease. On the day of discharge, 86% of the patients were aware of how their care and treatment after hospitalization would proceed. More than half (54%) perceived their health status as fair, related to their normal state of health.

### 5.2 Level of Patient‐Centred Quality of Nursing Care

The level of patient‐centred quality of nursing care was evaluated by patients in two ways: the Summative level of the quality of nursing care and the Quality of educational support provided to patients.

#### 5.2.1 Summative Level of the Quality of Nursing Care

Summative level of the quality of nursing care was evaluated by patients using two measures (Table 2, see also item‐level Supporting Table S1): Summative level of Good Nursing Care Scale (GNCS) and General Evaluation of the Quality of Nursing care (GEQN). Summative level of GNCS included 34 items and 6 subcategories (Nursing staff characteristics, Nursing care activities, Preconditions for care, Nursing environment, Proceeding of the nursing process and Patient empowerment). Summative level of the GNCS was evaluated as high (Median = 3.66, IQR = 0.73), internal consistency reliability being also high (*ω* = 0.98). Evaluations varied across the subcategories, between high and very high, highest being the subcategories of Nursing staff characteristics (Median = 3.80, IQR = 0.80) and Nursing environment (Median = 3.80, IQR = 0.60) and the lowest being Patient empowerment (Median = 3.29, IQR = 1.14).

**TABLE 2:** Level of quality of nursing care.

Good Nursing Care Scale (GNCS)^a^	*n*	Median (IQR)	Mean (SD)	Min‐ Max	*ω*
Summative level of GNCS	524	3.66 (0.73)	3.51 (0.45)	1.82–4.00	0.98
Subcategories of GNCS					
Nursing staff characteristics (5 items)	855	3.80 (0.80)	3.58 (0.48)	1.00–4.00	0.86
Nursing care activities (6 items)	825	3.60 (1.17)	3.37 (0.62)	1.00–4.00	0.90
Preconditions for care (5 items)	794	3.60 (1.00)	3.50 (0.50)	1.60–4.00	0.88
Nursing environment (5 items)	836	3.80 (0.60)	3.65 (0.43)	1.80–4.00	0.81
Proceeding of the nursing process (6 items)	771	3.50 (1.00)	3.35 (0.55)	1.60–4.00	0.89
Patient empowerment (7 items)	692	3.29 (1.14)	3.26 (0.61)	1.00–4.00	0.87
Collaboration with family members/significant others (6 items)	425	3.00 (1.27)	2.93 (0.84)	1.00–4.00	0.94

General Evaluation of Quality of Nursing care (GEQN)^b^	*n*	Median (IQR)	Mean (SD)	Min–Max
Quality of nursing care as a whole	855	9.00 (2.00)	8.84 (1.01)	4–10
Patient‐centredness	850	9.00 (2.00)	8.67 (1.13)	4–10
Fluency	854	9.00 (2.00)	8.66 (1.11)	4–10
Effectiveness	850	9.00 (2.00)	8.64 (1.18)	4–10
Competence of nurses	851	9.00 (1.00)	9.11 (0.92)	4–10

^a^ GNCS and subcategories: Four‐point Likert scale from 1 = strongly disagree to 4 = strongly agree, higher value indicating higher quality of nursing care. In the analysis, missing responses were allowed as follows: ≤ 1 item per sum variable; ≤ 2 sum variables with one missing item for GNCS summative level.

^b^ General evaluation of quality of nursing care: scale 4–10 (higher value indicating higher level of the quality).

The lowest evaluation, however, was in the subcategory of Collaboration with family members/significant others (Median = 3.00, IQR = 1.27). Almost all (99%) patients reported having family members/significant others, 90% of patients indicated that they would have wanted them to participate in their care, and 86% reported that they had participated.

General Evaluation of Quality of Nursing Care GEQN, measured with a numeral scale 4–10 (poor–excellent), was evaluated at a similar level across all dimensions, with the median being 9.00 (IQR 2.00–1.00). Internal consistency reliability of GEQN was high (*ω* = 0.94).

Correlations between the GNCS and GEQN were strong and statistically significant (Table 3). The weakest correlations were observed between the subcategory Collaboration with family members/significant others and all of the dimensions of GEON (0.27–0.43).

**TABLE 3:** Correlations between Good Nursing Care Scale (GNCS) and General Evaluation of Quality of Nursing Care (GEQN).

General Evaluation of Quality of Nursing care (GEQN)	Subcategories of Good Nursing Care Scale (GNCS)							
	Summative level of GNCS	Nursing staff characteristics	Nursing care activities	Preconditions for care	Nursing environment	Proceeding of the nursing process	Patient empowerment	Collaboration with family members/significant others
Quality or nursing care as a whole	0.63	0.55	0.58	0.56	0.48	0.53	0.52	0.35
Patient centredness	0.65	0.62	0.63	0.61	0.56	0.55	0.57	0.39
Fluency	0.65	0.62	0.62	0.63	0.55	0.56	0.57	0.40
Efficiency	0.66	0.61	0.62	0.62	0.54	0.60	0.57	0.43
Competence of nurses	0.60	0.61	0.56	0.57	0.51	0.53	0.54	0.27

*Note:* Spearman's rank‐order correlation. All correlations are statistically significant *p* < 0.05.

In addition to the results presented in Table 3, the Spearman's rank‐order correlation indicated that all GNCS subcategories were very strongly positively correlated (*r* = 0.83–0.9) with the Summative level of GNCS and strongly positively correlated (*r* = 0.63–0.81) with each other. The GNCS subcategory Patient empowerment evaluates patients' perspectives on their own empowerment. The association of this subcategory was also analysed with the Alternative GNCS total score, showing a strong positive correlation (*r* = 0.8) between these sums and indicating the important role of empowerment in the evaluation of the quality of nursing care.

#### 5.2.2 Quality of the Educational Support for Patients (EDUsupport)

The new summative variable, EDUsupport, is related to patient education as a nursing action, consisting of educational items from most of the GNCS subcategories, that is, deepening of the analysis of the quality of nursing care (Table 4). The EDUsupport total was high (Median = 3.46, IQR = 1.00), as were the levels for all individual items. The consistency of the EDUsupport was sufficient (*ω* = 0.95).

**TABLE 4:** Quality of educational support for patients (EDUsupport).

EDUsupport	*n*	Median (IQR)	Mean (SD)	Min–Max
EDUsupport total	600	3.46 (1.00)	3.32 (0.58)	1.38–4.00
Knowledgeable (Nursing staff characteristics)	854	4.00 (1.00)	3.28 (0.83)	1.00–4.00
Clarification of issues (Nursing care activities)	759	4.00 (1.00)	3.46 (0.73)	1.00–4.00
Self‐care support (Nursing care activities)	842	4.00 (2.00)	3.17 (0.97)	1.00–4.00
Educational (Nursing care activities)	852	3.00 (1.00)	3.19 (0.86)	1.00–4.00
Knowledge of professionals (Preconditions for care)	838	4.00 (1.00)	3.63 (0.52)	1.00–4.00
Evidence‐base (Preconditions for care)	680	4.00 (1.00)	3.63 (0.53)	1.00–4.00
Knowledge about discharge (Proceeding of the nursing process)	747	4.00 (1.00)	3.42 (0.71)	1.00–4.00
Knowledge about permissions to do (Proceeding of the nursing process)	814	3.00 (1.00)	3.26 (0.86)	1.00–4.00
Knowledge about complications at home (Proceeding of the nursing process)	818	3.00 (2.00)	3.14 (0.92)	1.00–4.00
Bio‐physiological (Patient empowerment)	830	4.00 (1.00)	3.52 (0.64)	1.00–4.00
Cognitive (Patient empowerment)	805	3.00 (2.00)	3.12 (0.86)	1.00–4.00
Economic (Patient empowerment)	703	3.00 (3.00)	2.59 (1.21)	1.00–4.00
Educational (Collaboration with family members/significant others)	624	3.00 (1.00)	3.22 (0.84)	1.00–4.00

*Note:* EDUsupport total and subcategories: Four‐point Likert type scale from 1 = strongly disagree to 4 = strongly agree, higher value indicating higher quality of nursing care. In the analysis missing responses were allowed as follows: ≤ 1 item per sum variable; ≤ 2 sum variables with one missing item for EDUsupport total summative level.

### 5.3 Factors Associated With the Summative Level of the Quality of Nursing Care GNCS

Three statistically significant associations were found between the background factors and the Summative level of the GNCS (Table 5). The first association was sufficient information prior to hospitalization (*p* < 0.001, adjusted *p* = 0.002, *r* = 0.167, 95% CI = (0.080–0.250)) patients who received sufficient information prior to hospitalization evaluated the Summative level of GNCS higher than those who did not receive sufficient information. Sufficient information prior to hospitalization also had a statistically significant association with the following GNCS subcategories: Patient empowerment (*p* < 0.001, adjusted *p* < 0.001, *r* = 0.177, 95% CI = (0.100–0.250)), Nursing staff characteristics (*p* < 0.001, adjusted *p* < 0.001, *r* = 0.136, 95% CI = (0.070–0.200)), Nursing care activities (*p* < 0.001, adjusted *p* < 0.001, *r* = 0.161, 95% CI = (0.100–0.230)), Preconditions for care (*p* < 0.001, adjusted *p* < 0.001, *r* = 0.156, 95% CI = (0.090–0.230)), Proceeding of the nursing process (*p* = 0.007, adjusted *p* = 0.0140) and Nursing environment (*p* = 0.009, adjusted *p* = 0.0140, *r* = 0.099, 95% CI = (0.030–0.170)). Sufficient information prior to hospitalization had a positive association in the quality of nursing care in these subcategories. This association remained also in linear regression for the Summative level of the GNCS (*β* = 0.135, 95% CI = (0.033, 0.237), standardized *β* = 0.30, 95% CI = (0.07, 0.53)) (Supporting Table S3).

**TABLE 5:** Background factors and Summative level of Good Nursing Care Scale (GNCS).

Background factor	*n*	Median (IQR)	Mean (SD)	Spearman's *ρ*	*p*	Adjusted *p*	Effect size (95% CI)^a^
Age				0.166			
Gender							
Male	282	3.65 (0.78)	3.50 (0.46)		0.785	1.000	0.0118 (0.0014–0.1)
Female	254	3.68 (0.68)	3.51 (0.44)				
Does not want to disclosure^b^	2						
Education							
Comprehensive/secondary education	366	3.66 (0.76)	3.51 (0.45)		0.317	1.000	0.0436 (0.0018–0.13)
University/Polytechnic/Doctoral degree	161	3.65 (0.71)	3.48 (0.45)				
Work status							
Not working	366	3.70 (0.73)	3.53 (0.45)		0.022	0.245	0.0982 (0.02–0.18)
Working	176	3.55 (0.71)	3.45 (0.45)				
Living arrangement							
Live alone	196	3.62 (0.92)	3.46 (0.49)		0.257	1.000	0.0489 (0.0023–0.13)
Live with family members/significant others	341	3.68 (0.61)	3.53 (0.42)				
Unit							
Medical	185	3.70 (0.76)	3.53 (0.43)		0.271	1.000	0.0484 (0.0019–0.14)
Operational	334	3.64 (0.76)	3.48 (0.46)				
Type of admission into hospital							
Emergency patient	304	3.65 (0.76)	3.49 (0.45)		0.268	1.000	0.0480 (0.0028–0.14)
Elective patient	230	3.68 (0.70)	3.52 (0.45)				
Time of information of admission into hospital							
Emergency	300	3.65 (0.76)	3.49 (0.45)		0.267	1.000	0.0003 (−0.0012–0.0097)^a^
1–3 days	19	3.68 (0.97)	3.53 (0.48)				
4–10 days	28	3.65 (0.72)	3.44 (0.45)				
11–30 days	50	3.69 (0.53)	3.59 (0.42)				
Over a month	116	3.71 (0.66)	3.53 (0.45)				
First time at hospital generally							
Yes	56	3.44 (0.91)	3.39 (0.48)		0.084	0.747	0.0747 (0.0041–0.16)
No	480	3.66 (0.71)	3.52 (0.44)				
Received sufficient information prior to hospitalization							
No	129	3.45 (0.73)	3.38 (0.46)		< 0.001	0.002	0.1670 (0.08–0.25)
Yes	398	3.71 (0.73)	3.54 (0.45)				
Knowledge about proceeding of care and treatment							
No	57	3.68 (0.59)	3.53 (0.41)		0.809	1.000	0.0104 (0.0012–0.09)
Yes	480	3.65 (0.76)	3.50 (0.45)				
Days spent in this hospital							
<= 2	162	3.66 (0.67)	3.50 (0.46)		0.960	1.000	−0.0031 (−0.0033 to 0.0083)^a^
3–5	230	3.66 (0.70)	3.52 (0.43)				
6–10	87	3.59 (0.77)	3.49 (0.44)				
11 <	40	3.69 (0.86)	3.47 (0.50)				
Any long‐term disease							
No	154	3.67 (0.75)	3.49 (0.46)		0.955	1.000	0.0024 (0.0014–0.11)
Yes	384	3.65 (0.74)	3.51 (0.45)				
Current health status in comparison with normal state of health							
Excellent/good	201	3.76 (0.72)	3.56 (0.46)		< 0.001	0.011	0.1440 (0.05–0.23)
Fair/poor	333	3.59 (0.71)	3.47 (0.44)				

*Note:* Effect sizes are reported as *r* for Mann–Whitney *U* tests; 95% CI calculated using bootstrapping.

^a^ *η* ^2^ for Kruskal–Wallis tests.

^b^ Not included in testing.

The second statistically significant background factor was the patient's current health status on the day of discharge, compared with their normal health status. Patients with a fair or poor state of health at the end of their hospital process (outcome of care) evaluated the Summative level of GNCS significantly lower (*p* < 0.001, adjusted *p* = 0.011, *r* = 0.144, 95% CI = (0.050–0.230)) than patients with a good or excellent health status (Table 5). A significant association was observed between fair/poor health and only one of the GNCS subcategories, Proceeding of the nursing process (*p* = 0.004, adjusted *p* = 0.024, *r* = 0.106, 95% CI = (0.040–0.170)). Patients with a fair or poor state of health evaluated the subcategory of Proceeding of the nursing process as lower than those with better health status.

The third statistically significant association with the Summative level of the GNCS was the patient's work status. Patients who were not working evaluated the quality of nursing care as significantly higher (*p* = 0.022, *r* = 0.098, 95% CI = (0.020–0.180)) than those working; however, this association did not remain after Holm–Bonferroni correction (adjusted *p* = 0.245). No significant associations between the GNCS subcategories and patients' work status were observed.

No statistically significant association with background factors and the sum variable Collaboration with family members/significant others was found (see Supporting Table S2).

In the analysis of EDUsupport and background factors, two factors were tested: Education of the patient and Received sufficient information prior to hospitalization (Table 6). There was no statistically significant association between Education of the patient and EDUsupport. However, an association was found between Received sufficient information prior to hospitalization and EDUsupport (*p* < 0.001, *r* = 0.166, 95% CI = (0.090–0.240)): patients who had received sufficient information prior to hospitalization evaluated the EDUsupport as higher than those who did not receive sufficient information.

**TABLE 6:** Background factors and EDUsupport.

Variable	*n*	Median (IQR)	Mean (SD)	*p*	Effect size (95% CI)
Received sufficient information prior to hospitalization					
No	142	3.23 (0.91)	3.17 (0.55)	< 0.001	0.166 (0.09–0.24)
Yes	439	3.54 (1.07)	3.36 (0.58)		
Education					
Comprehensive/Secondary education	404	3.50 (1.03)	3.33 (0.58)	0.255	0.0471 (0.0027–0.12)
University/ Polytechnic/ Doctoral degree	179	3.45 (0.92)	3.29 (0.56)		

*Note:* Effect sizes are reported as *r*. 95% CI calculated using bootstrapping.

## 6 Discussion

### 6.1 Discussion About Results

The focus of this study, patient‐centred quality of nursing care, is both timely and important, given the challenges in health care and the central role of patients' experience‐based measures in quality assurance. The study both confirms earlier knowledge and gives novel findings. The main finding of this study clearly indicates a high level of nursing care quality, as evaluated by patients themselves in hospital care, consistent also with earlier research (Havana et al. 2023). The result is consistent also with earlier findings using Good Nursing Care Scale across international and diverse clinical contexts (Mattila et al. 2025), other evaluative instruments (Guzmán‐Leguel and Rodríguez‐Lara 2025), and earlier reports (OECD and European Observatory on Health Systems and Policies 2025). However, the existence of several instruments with differing theoretical conceptualizations of nursing care quality (Friedel et al. 2023) limits the generalization of these results. Our definition of nursing—based on theory on human action—emphasizes the association between multidimensional theoretical and clinical/practical knowledge. However, although participants reported high levels of quality of nursing care, the interpretation of these findings should be cautious because of the possible ceiling effects that may have led to overly positive ratings.

The study contributes also new evidence related to the quality of nursing care. Variation in the level of nursing care quality was noticed in the subcategories of the GNCS, with the highest ratings in Nursing environment and the lowest in Collaboration with family members/significant others. Collaboration with family members has been evaluated even lower in earlier research (Mattila et al. 2025). This can be explained by the fact that patients were not necessarily aware of the collaboration between the nurses and their family members/significant others. Further, a substantially lower number of patients (*n* = 425) responded to this subcategory compared to other subcategories and the correlation with the other categories was lower. For these reasons, this subcategory was not included in the analysis in the Summative level of GNCS, highlighting the need to analyse this subcategory in detail in future and reformulate this aspect of the GNCS. This subcategory is important for the realization of the quality of patient‐centred nursing care, especially in connection with the increasing remote services in health care requiring new role also for significant others.

Specific observations can be made regarding the subcategory Patient empowerment and the new summative variable Educational support (EDUsupport). Both were rated highly. The subcategory Patient empowerment was also analysed in relation to the Alternative Summative level of GNCS (including all subcategories except Patient Empowerment), and a strong correlation was found. Thus, in the future, patient empowerment could be considered a theoretically relevant outcome of nursing care quality. Empowerment can be seen as a process and/or outcome. In this study, it is one category of the quality, expressing mainly the experienced support of empowerment for patients. A more systematic combination of empowerment as a process and outcome could be the new addition to the Good Nursing Care Scale.

The new summative variable EDUsupport represents a novelty in this study as it reflects the quality of nursing care as well as patients' rights (Inkeroinen et al. 2025). The level of educational support was high in this study, financial knowledge being the area for development (Arffman et al. 2025). This is an important finding, linking the patients' experience‐based evaluations and social determinants of health (Attar and Shukla 2025; WHO 2025). In the future, the financial element of the quality of nursing care should be stronger, also included in the Good Nursing Care Scale.

### 6.2 Factors Associated With the Quality of Nursing Care

Three important factors related to the quality of nursing care were identified, although low effect sizes limited clinical relevance: Received sufficient information prior to hospitalization, health status on the day of discharge and patients' work status. Information prior to hospitalization had the strongest association with the quality of nursing care, the only one staying in the linear regression. This information can support the orientation of the patients and align their expectations with the knowledge they actually receive, as patients often seem to expect more than they receive (Arffman et al. 2025). This finding highlights the need to systematize the educational support for patients, including a timely orientation to hospital care. Overall, patients seemed to be well prepared for the discharge from hospital.

Health status of the patient on the day of discharge has special importance, because it can express the potential outcome (PROMS) of the quality of nursing care. In this study, patients with lower health status evaluated the quality of nursing care lower, especially in the subcategory of Proceeding of the nursing process. Future research should examine in more detail the association between health status and quality of nursing care throughout the entire process, and also including intervention studies. This is especially important given the recently identified self‐reported unmet needs in access to services in Finland (OECD and European Observatory on Health Systems and Policies 2025).

Work status of patients also earns further analysis. In this study, the first finding was that patients not in worklife evaluated the quality higher than others. The association, however, did not remain after the Holm–Bonferroni correction. In future, the factor of work status may have specific importance due to changes in sociodemographics of the population, including the growing number of older people and those not in worklife.

### 6.3 Strengths and Limitations of the Study

This study has several strengths and limitations. In the cross‐sectional design, the use of a valid, internationally tested instrument, Good Nursing Care Scale (Leino‐Kilpi2025) (Mattila et al. 2025; Stolt et al. 2019), can be considered a strength, also having connection with the conceptual basis of the study. The scale is useable for patients, allows assessment of a multidimensional concept of the patient‐centred quality of nursing care, has a rather high response rate, offers an option between paper‐and‐pencil and online participation and has demonstrated high internal consistency. The scale could be enhanced with added content related to the outcomes of care (PROMS) and the multidimensional nature of patient empowerment. Consideration could also be given to shortening the scale due to the strong correlations between Summative level of GNCS, its subcategories and the General Evaluation of the Quality of Nursing care (GEQN). This should be interpreted with caution because a conceptual overlap between the GNCS and GEQN may contribute to the high correlations. These findings suggest that evaluation of the level of the quality of nursing care could be made with lower number of items. In its current form, however the GNCS produces multidimensional knowledge of the quality of nursing care and can be recommend for overall evaluation and for different elements of nursing care. The cross‐sectional design of this study aimed to produce actual evaluative data, but it could be supported by observing nursing actions, testing the causality between the actions and outcomes in interventional designs and comparing the results using different quality evaluation instruments (e.g., Correia et al. 2025; Friedel et al. 2023).

The study sample represents both a strength and a limitation. As a strength, there were a high number of respondents, exceeding the requirement indicated by the power analysis, and the length of hospitalization being typical for the country (OECD 2025b). However, the number of patients who declined to participate is unknown due to the heavy workload in the units. This can mean that those having positive experiences and evaluating the quality higher have been more interested to respond. In the analysis, few statistically significant background factors of patients associated with the level of quality were identified. The results should be generalized with caution, especially because the number and nature of declining patients is not known. Another limitation concerns sample representativeness: the study included two public wellbeing services counties and their main hospitals, which are part of the Finnish public health care and social welfare system, under the administration of the Ministry of Social and Health Services, and have the same duties related to population. However, the counties are part of a rather recently (since 2023) started reform in health care and social affairs, and they differ in population size and local arrangements due to the ongoing reform. Therefore, comparisons between the counties were not relevant. In future studies, additional organizational factors could be included in the analysis. The leadership strategies of the quality of nursing care (Abugre and Bhengu 2024; Linnavuori et al. 2024) would be especially important.

The significance of patients' age, educational background, living arrangements, work status, hospital admission and length of hospital stay can vary between studies (Guan et al. 2024; Mattila et al. 2025; Volakakis et al. 2024). In contrast, good health status on the day of discharge and knowledge about one's care have been more reliably identified as elements developing quality of care (Mattila et al. 2025). Experienced quality also seems to be associated with national health expenditure per capita as well as other factors beyond the health system (OECD 2025a). Thus, there is still a need for analyses about the associations of background factors with the quality, also using randomized samples and longitudinal studies.

Another limitation of the study is the reliance on self‐evaluation. Patients may evaluate the quality of their nursing care more positively than objective measures would suggest for a variety of reasons, for example, due to access to care and treatment, trust and respect for professionals, unwillingness to complain and a tendency to respond in a socially desirable way (Badejo et al. 2022; Havana et al. 2023). In this study, patient responses were considered the best measure of their care process and the quality they experienced. It would be valuable for future research to combine self‐evaluation with other methods, for example register‐based data including clinical outcome measures, process measures and structural measures, thereby combining multiple factors of quality and the resources that support them. Collecting survey responses on the day of discharge may induce social desirability bias. Patients may be reluctant to express negative opinions about their care because they wish to maintain positive relationships with healthcare professionals, feel grateful for the care received, or fear that critical responses could affect their ongoing care or discharge process.

Incomplete responses warrant specific consideration because they required inclusion in the analysis under certain criteria. Good Nursing Care Scale includes the possibility of responding with ‘I cannot evaluate’, and although such responses may reflect meaningful uncertainty or lack of knowledge, they do not clearly inform the underlying latent construct measured by the scale and were therefore treated as missing in the analyses. To balance data retention with measurement integrity, a limited amount of missingness was permitted. This approach may underrepresent those who expressed greater uncertainty, potentially introducing bias in scale scores or associations. However, imputing uncertain responses would require imposing an artificial position on the Likert continuum and could introduce bias. Future research could examine ‘I cannot evaluate’ responses as a distinct outcome, as their frequency may carry substantive information about respondent knowledge, experience or construct applicability. It is also relevant to examine which items are likely to result in these responses and the reasons for that. In this study, over 30% of patients responded with ‘I cannot evaluate’ in subcategory Collaboration with family members/significant others, specifically for the items on encouragement, reservation of time and listening, as well as in the subcategory Patient empowerment, the item experiential. Evaluating the experiences of others is understandably more difficult, and some patients may not be aware of care‐related discussions, even if their relatives have been involved.

As a summary, this study has both strengths and limitations. It provides strong evidence of the high quality of nursing care experienced by hospitalized patients. This is an important finding, as the data were collected rather close to the time of Covid19 and a large reform of social and health care in the country. Thus, the level of quality earns continuous systematic evaluative procedures and testing different practices related to quality.

### 6.4 Recommendations for Further Research

This study provides several recommendations for further research, which can be divided into conceptual, methodological and clinical areas. Conceptually, the action‐based definition used in the Good Nursing Care Scale could be compared by using instruments with different conceptualizations for producing comparative knowledge in the field. The results support the use of the Good Nursing Care Scale in the analysis of patient‐centred quality of nursing care, but patient empowerment as a nursing outcome warrants further investigation, as does the subcategory of Collaboration with family members and significant others.

Another recommendation for future research is to examine patients' own contributions to the quality of nursing care. Patients not only receive care but also play a role in co‐designing and developing care. Including this perspective in future studies could provide new insights into quality evaluation and support the construct of systematic procedures for monitoring, evaluation, and patients' rights and duties. Family members and significant others should also be involved, although their role was not clear in the results of this study. In future, their role can also change due to changes in health care.

Methodologically, future research should evaluate the quality of nursing care in randomized patient samples, with added potential factors such as social determinants of health (WHO 2025), details of the proceeding of nursing process, associations between hospital and primary health care, and factors of nursing workforce competence and resources. Furthermore, the associations between patient‐experienced (PREMS) and patient‐outcomes (PROMS) should be analysed and tested in interventional or longitudinal studies. International comparisons of the patient‐centred quality of nursing care and associated factors could provide a good basis for developing nursing care globally. Related to the newly constructed scale of EDUSupport, further validity testing is also needed.

Clinically, educational support should be systematically analysed to identify the best practices for empowering patients. This study indicates that educational support is multidimensional and relevant for different parts of nursing action and that the new construct of EDUsupport should be tested and systematized in diverse clinical fields. It is particularly important to evaluate new constructions of information prior to hospitalization.

Finally, future studies should investigate the factors contributing to the currently high‐level patient‐centred quality of nursing care on a broad level. Are these outcomes the result of the competence of nurses and/or administrative actions, strategies of health care or new technological solutions and how sustainable are they? Examining these factors would allow us to establish a comprehensive understanding of evaluations of nursing care quality across micro‐, meso‐ and macro‐level contexts.

## 7 Conclusion

The patient‐centred quality of nursing care, as evaluated by hospital patients in Finland, was found to generally be at a high level. Nevertheless, developments are needed in educational elements—such as orientation to hospital care and financial information—collaboration with significant others and the association between quality of nursing care and patient outcomes. Future research should also address the organizational‐level factors aiming to develop the association between patient‐centred experiences (PREMS) and outcome measures (PROMS), to establish a larger understanding of the patient‐centred quality of nursing care.

## Author Contributions

**Helena Leino‐Kilpi:** conceptualization, funding acquisition, investigation, methodology, project administration, writing – review and editing, writing – original draft. **Sanna Mäkinen:** visualization, writing – original draft, writing – review and editing. **Saija Inkeroinen:** visualization, writing – review and editing. **Miko Pasanen:** formal analysis, writing – original draft, writing – review and editing. **Jenna Poraharju:** resources, writing – review and editing, visualization. **Heli Virtanen:** writing – review and editing. **Minna Stolt:** methodology, writing – review and editing.

## Funding

This work was supported by Wellbeing Services County of Southwest Finland (13762).

## Ethics Statement

Ethical approval was granted by the Ethics Committee for Human Sciences at the University of Turku, Health Care Division (TY/253/2025). Research Approvals: obtained from both wellbeing services counties (County 1: 27th of March 2025 and County 2: 24th of April 2025). Copyright‐holder of the GNCS‐instrument (Good Nursing Care Scale Leino‐Kilpi 2025) is a member of the research team and the team has the right to use it. There is a statistician on the research team and among authors (Miko Pasanen). Additional references have been included at the reviewers' request.

## Conflicts of Interest

The authors declare no conflicts of interest.

## Supporting information


**Table S1:** Structure and content of Good Nursing Care Scale (GNCS Leino‐Kilpi 2025) and quality of nursing care.

**Table S2:** Background factors and Collaboration with family members/significant others.

**Table S3:** Linear regression of Summative level of Good Nursing Care Scale (GNCS) and factors related to the process of care and health of the patient.

## Data Availability

Research data are not shared.
