# Supplementary material for: Patient‐Centred Quality of Nursing Care: A Cross‐Sectional Study of the Level and Associated Factors as Perceived by Patients
Source: Nurs Open. 2026 Nov 1;13(11):e70995. doi: 10.1002/nop2.70995 (PMC13724307; doi:10.1002/nop2.70995)
Supplement: Supplementary file 1 [file NOP2-13-e70995-s001.docx]

Supporting Table S2. Background factors and Collaboration with family members/significant others

| Background factor | | n | Median (IQR) | Mean (SD) | Spearman’s ρ | p | Adjusted p | Effect size (95% CI)* | |
| --- | --- | --- | --- | --- | --- | --- | --- | --- | --- |
| Age |  |  |  |  | 0.087 |  | | |  |
|  |  |  |  |  |  |  | | |  |
| Gender |  |  |  |  |  |  | | |  |
|  | Male | 218 | 3 .00 (1.43) | 2.95 (0.84) |  |  |  |  | |
|  | Female | 203 | 3 .00 (1.10) | 2.89 (0.84) |  |  |  |  | |
|  | Does not want to disclosure ** | 0 |  |  |  |  |  |  | |
|  |  |  |  |  |  | 0.557 | 1.000 | 0.0286 (0.0019-0.13) | |
| Education | |  |  |  |  |  |  |  | |
|  | Comprehensive/  secondary education | 312 | 3.00 (1.17) | 2.95 (0.81) |  |  |  |  | |
|  | University/  polytechnic/  doctoral degree | 101 | 3.00 (1.33) | 2.83 (0.92) |  |  |  |  | |
|  |  |  |  |  |  | 0.428 | 1.000 | 0.0390 (0.002-0.13) | |
| Work status | |  |  |  |  |  |  |  | |
|  | Not working | 305 | 3.00 (1.17) | 2.97 (0.82) |  |  |  |  | |
|  | Working | 120 | 2.83 (1.33) | 2.82 (0.87) |  |  |  |  | |
|  |  |  |  |  |  | 0.091 | 1.000 | 0.0820 (0.0055-0.18) | |
| Living arrangement | |  |  |  |  |  |  |  | |
|  | Live alone | 135 | 2.83 (1.17) | 2.84 (0.84) |  |  |  |  | |
|  | Live with family members/ significant others | 286 | 3.00 (1.41) | 2.96 (0.84) |  |  |  |  | |
|  |  |  |  |  |  | 0.149 | 1.000 | 0.0704 (0.0048-0.16) | |
| Unit |  |  |  |  |  |  |  |  | |
|  | Medical | 145 | 3.00 (1.27) | 2.94 (0.85) |  |  |  |  | |
|  | Operational | 269 | 2.83 (1.27) | 2.90 (0.84) |  |  |  |  | |
|  |  |  |  |  |  | 0.571 | 1.000 | 0.0279 (0.0017-0.13) | |
| Type of admission into hospital | |  |  |  |  |  |  |  | |
|  | Emergency patient | 242 | 3.00 (1.27) | 2.89 (0.87) |  |  |  |  | |
|  | Elective patient | 176 | 3.00 (1.27) | 2.96 (0.79) |  |  |  |  | |
|  |  |  |  |  |  | 0.722 | 1.000 | 0.0174 (0.0016-0.11) | |
| Time of information of  admission into hospital | |  |  |  |  |  |  |  | |
|  | Emergency | 238 | 3.00 (1.23) | 2.88 (0.87) |  |  |  |  | |
|  | 1-3 days | 12 | 3.33 (0.88) | 3.31 (0.55) |  |  |  |  | |
|  | 4-10 days | 20 | 3.00 (1.13) | 3.01 (0.77) |  |  |  |  | |
|  | 11-30 days | 38 | 2.82 (1.12) | 2.79 (0.84) |  |  |  |  | |
|  | Over a month | 89 | 2.83 (1.50) | 2.92 (0.83) |  |  |  |  | |
|  |  |  |  |  |  | 0.722 | 1.000 | -0.0010 (-0.0012-0.0052) | |
| First time at hospital generally | |  |  |  |  |  |  |  | |
|  | Yes | 48 | 2.83 (1.04) | 2.76 (0.84) |  |  |  |  | |
|  | No | 371 | 3.00 (1.27) | 2.94 (0.84) |  |  |  |  | |
|  |  |  |  |  |  | 0.175 | 1.000 | 0.0663 (0.0033-0.16) | |
| Received sufficient information prior to hospitalization | |  |  |  |  |  |  |  | |
|  | No | 99 | 2.83 (1.13) | 2.77 (0.91) |  |  |  |  | |
|  | Yes | 312 | 3.00 (1.27) | 2.96 (0.82) |  |  |  |  | |
|  |  |  |  |  |  | 0.108 | 1.000 | 0.0794 (0.0058-0.17) | |
| Knowledge about proceeding of care and treatment | |  |  |  |  |  |  |  | |
|  | No | 59 | 3.17 (1.03) | 3.02 (0.85) |  |  |  |  | |
|  | Yes | 363 | 3.00 (1.27) | 2.92 (0.83) |  |  |  |  | |
|  |  |  |  |  |  | 0.262 | 1.000 | 0.0547 (0.0032-0.15) | |
| Days spent in this hospital | |  |  |  |  |  |  |  | |
|  | <=2 | 124 | 3.00 (1.38) | 2.88 (0.89) |  |  |  |  | |
|  | 3-5 | 168 | 3.00 (1.12) | 2.89 (0.84) |  |  |  |  | |
|  | 6-10 | 79 | 3.00 (1.17) | 3.02 (0.77) |  |  |  |  | |
|  | 11< | 30 | 3.00 (1.25) | 3.06 (0.75) |  |  |  |  | |
|  |  |  |  |  |  | 0.677 | 1.000 | -0.0017 (-0.0031-0.01) | |
| Any long-term disease | |  |  |  |  |  |  |  | |
|  | No | 112 | 3.00 (1.26) | 3.00 (0.86) |  |  |  |  | |
|  | Yes | 310 | 2.83 (1.18) | 2.89 (0.83) |  |  |  |  | |
|  |  |  |  |  |  | 0.133 | 1.000 | 0.0731 (0.004-0.16) | |
| Current health status in comparison with normal state of health | |  |  |  |  |  |  |  | |
|  | Excellent/ good | 146 | 3.00 (1.43) | 3.03 (0.84) |  |  |  |  | |
|  | Fairly good /poor | 274 | 2.83 (1.17) | 2.87 (0.84) |  |  |  |  | |
|  |  |  |  |  |  | 0.052 | 0.676 | 0.0950 (0.01-0.19) | |

Effect sizes are reported as r for Mann–Whitney U tests and *η² for Kruskal–Wallis tests. 95%-CI calculated using bootstrapping. ** Not included in testing.
