# Supplementary material for: Patient‐Centred Quality of Nursing Care: A Cross‐Sectional Study of the Level and Associated Factors as Perceived by Patients
Source: Nurs Open. 2026 Nov 1;13(11):e70995. doi: 10.1002/nop2.70995 (PMC13724307; doi:10.1002/nop2.70995)
Supplement: Supplementary file 2 [file NOP2-13-e70995-s002.docx]

Supporting Table S3. Linear regression of Summative level of Good Nursing Care -scale (GNCS) and factors related to the process of care and health of the patient

|  | B | SE | 95% CI | β | 95% CI β |
| --- | --- | --- | --- | --- | --- |
| Intercept | 3.357 | 0.108 | 3.145 – 3.570 | -0.331 | -0.806 – 0.144 |
| Received sufficient information prior to hospitalization: No vs. Yes | 0.135 | 0.052 | **0.033 – 0.237** | 0.302 | **0.074 – 0.529** |
| Type of admission into hospital: Emergency vs. Elective | 0.005 | 0.047 | -0.087 – 0.097 | 0.011 | -0.194 – 0.216 |
| First time at hospital: Yes vs. No | 0.105 | 0.067 | -0.027 – 0.237 | 0.235 | -0.060 – 0.530 |
| Knowledge about proceeding of care and treatment: No vs. Yes | -0.034 | 0.066 | -0.164 – 0.097 | -0.075 | -0.366 – 0.216 |
| Days spent in this hospital: <=2 vs. 3-5 | 0.045 | 0.049 | -0.052 – 0.141 | 0.099 | -0.116 – 0.315 |
| Days spent in this hospital: <=2 vs. 6-10 | 0.016 | 0.065 | -0.112 – 0.143 | 0.035 | -0.250 – 0.320 |
| Days spent in this hospital: <=2 vs. 11< | 0.010 | 0.085 | -0.157 – 0.176 | 0.022 | -0.350 – 0.394 |
| Any long-term disease: No vs. Yes | 0.008 | 0.045 | -0.081 – 0.097 | 0.017 | -0.181 – 0.216 |
| Current health status in comparison with normal state of health Excellent/good vs. Fair/ poor | -0.078 | 0.042 | -0.160 – 0.005 | -0.173 | -0.358 – 0.012 |

R^2^ = 0.034, Adjusted R^2^ = 0.015
