# Supplementary material for: Patient‐Centred Quality of Nursing Care: A Cross‐Sectional Study of the Level and Associated Factors as Perceived by Patients
Source: Nurs Open. 2026 Nov 1;13(11):e70995. doi: 10.1002/nop2.70995 (PMC13724307; doi:10.1002/nop2.70995)
Supplement: Supplementary file 3 [file NOP2-13-e70995-s003.docx]

Supporting Table S1. Structure and content of Good Nursing Care Scale (GNCS ©Leino-Kilpi2025) and quality of nursing care

| GNCS* | Item | n | Median (IQR) | Mean (SD) | Min- Max | I cannot evaluate (% |
| --- | --- | --- | --- | --- | --- | --- |
| **Summative level of GNCS** | | 524 | 3.66 (0.73) | 3.51 (0.45) | 1.82- 4.00 |  |
| **Subcategories of GNCS** | |  |  |  |  |  |
| *Nursing staff characteristics*  (5 items) | | 855 | 3.80 (0.80) | 3.58 (0.48) | 1.00- 4.00 |  |
|  | Kindness | 857 | 4.00 (0.00) | 3.76 (0.46) | 1.00- 4.00 | 0.12 |
|  | Carefulness | 857 | 4.00 (1.00) | 3.61 (0.55) | 1.00- 4.00 | 0.23 |
|  | Knowledgeable | 854 | 4.00 (1.00) | 3.28 (0.83) | 1.00- 4.00 | 0.35 |
|  | Service-orientation | 852 | 4.00 (1.00) | 3.58 (0.65) | 1.00- 4.00 | 0.81 |
|  | Honesty | 780 | 4.00 (1.00) | 3.73 (0.48) | 1.00- 4.00 | 8.88 |
| *Nursing care activities*  (6 items) | | 825 | 3.60 (1.17) | 3.37 (0.62) | 1.00- 4.00 |  |
|  | Educational | 852 | 3.00 (1.00) | 3.19 (0.86) | 1.00- 4.00 | 0.70 |
|  | Professional skills | 852 | 4.00 (1.00) | 3.67 (0.51) | 1.00- 4.00 | 0.70 |
|  | Self-care support | 842 | 4.00 (2.00) | 3.17 (0.97) | 1.00- 4.00 | 1.52 |
|  | Listening | 853 | 4.00 (1.00) | 3.50 (0.65) | 1.00- 4.00 | 0.47 |
|  | Clarification of issues | 759 | 4.00 (1.00) | 3.46 (0.73) | 1.00- 4.00 | 10.81 |
|  | Encouragement | 807 | 3.00 (1.00) | 3.27 (0.83) | 1.00- 4.00 | 5.61 |
| *Preconditions for care*  (5 items) | | 794 | 3.60 (1.00) | 3.50 (0.50) | 1.60- 4.00 |  |
|  | Knowledge of professionals | 838 | 4.00 (1.00) | 3.63 (0.52) | 1.00- 4.00 | 2.22 |
|  | Evidence-base | 680 | 4.00 (1.00) | 3.63 (0.53) | 1.00- 4.00 | 20.28 |
|  | Resources needed | 831 | 4.00 (1.00) | 3.31 (0.82) | 1.00- 4.00 | 2.81 |
|  | Prioritization of patient’s good | 795 | 3.00 (1.00) | 3.27 (0.81) | 1.00- 4.00 | 6.36 |
|  | Professional experience | 820 | 4.00 (1.00) | 3.68 (0.50) | 1.00- 4.00 | 3.87 |
| *Nursing environment*  (5 items) | | 836 | 3.80 (0.60) | 3.65 (0.43) | 1.80- 4.00 |  |
|  | Physical safety | 857 | 4.00 (1.00) | 3.67 (0.53) | 1.00- 4.00 | 0.00 |
|  | Psychological safety | 850 | 4.00 (1.00) | 3.42 (0.82) | 1.00- 4.00 | 0.58 |
|  | Aseptics | 823 | 4.00 (0.00) | 3.75 (0.46) | 1.00- 4.00 | 3.86 |
|  | Correct medication | 826 | 4.00 (0.00) | 3.76 (0.49) | 1.00- 4.00 | 3.28 |
|  | Confirmation of identity | 799 | 4.00 (0.00) | 3.67 (0.68) | 1.00- 4.00 | 6.77 |
| *Proceeding of the nursing process*  (6 items) | | 771 | 3.50 (1.00) | 3.35 (0.55) | 1.60- 4.00 |  |
|  | Access to care | 847 | 4.00 (1.00) | 3.50 (0.79) | 1.00- 4.00 | 0.94 |
|  | Collaboration with units | 693 | 4.00 (1.00) | 3.34 (0.78) | 1.00- 4.00 | 17.70 |
|  | Sufficient length of staying | 835 | 4.00 (1.00) | 3.49 (0.72) | 1.00- 4.00 | 2.11 |
|  | Knowledge about discharge | 747 | 4.00 (1.00) | 3.42 (0.71) | 1.00- 4.00 | 11.18 |
|  | Knowledge about complications at home | 818 | 3.00 (2.00) | 3.14 (0.92) | 1.00- 4.00 | 3.76 |
|  | Knowledge about permissions to do | 814 | 3.00 (1.00) | 3.26 (0.86) | 1.00- 4.00 | 4.01 |
| *Patient empowerment*  (7 items) | | 692 | 3.29 (1.14) | 3.26 (0.61) | 1.00- 4.00 |  |
|  | Experiential | 563 | 3.00 (1.00) | 3.23 (0.85) | 1.00- 4.00 | 32.90 |
|  | Cognitive | 805 | 3.00 (2.00) | 3.12 (0.86) | 1.00- 4.00 | 5.29 |
|  | Functional | 800 | 3.00 (1.00) | 3.31 (0.75) | 1.00- 4.00 | 5.77 |
|  | Ethical | 805 | 3.00 (1.00) | 3.35 (0.70) | 1.00- 4.00 | 4.73 |
|  | Social | 800 | 4.00 (1.00) | 3.54 (0.68) | 1.00- 4.00 | 5.99 |
|  | Economic | 703 | 3.00 (3.00) | 2.59 (1.21) | 1.00- 4.00 | 16.71 |
|  | Bio-physiological | 830 | 4.00 (1.00) | 3.52 (0.64) | 1.00- 4.00 | 2.47 |
| *Collaboration with family members/significant others*  (6 items) | | 425 | 3.00 (1.27) | 2.93 (0.84) | 1.00- 4.00 |  |
|  | Educational | 624 | 3.00 (1.00) | 3.22 (0.84) | 1.00- 4.00 | 13.57 |
|  | Participation in planning care | 596 | 3.00 (1.00) | 3.07 (0.85) | 1.00- 4.00 | 16.76 |
|  | Participation in evaluation of care | 573 | 2.00 (1.00) | 2.47 (1.09) | 1.00- 4.00 | 19.75 |
|  | Listening | 471 | 3.00 (2.00) | 2.96 (0.99) | 1.00- 4.00 | 33.85 |
|  | Encouragement | 409 | 3.00 (2.00) | 2.76 (1.08) | 1.00- 4.00 | 42.64 |
|  | Reservation of time | 460 | 3.00 (2.00) | 2.85 (1.04) | 1.00- 4.00 | 35.39 |

*Four-point Likert type scale: strongly agree- somewhat agree- somewhat disagree- strongly disagree and I don’t know. The higher value, the higher quality of nursing care. The interpretation related to the level of the quality: 1-1.5= very low quality, 1.6-2.0= low quality, 2,1-2,5= fairly low quality, 2,6-3,0=fairly high quality, 3,1-3,5= high quality and 3,6.4,0 =very high quality of care. In the analysis missing responses were allowed as follows: ≤1 item per sum variable; ≤2 sum variables with one missing item for summative level of GNCS.
